# Passive, yet not inactive: robotic exoskeleton walking increases cortical activation dependent on task

**DOI:** 10.1186/s12984-020-00739-6

**Published:** 2020-08-10

**Authors:** Sue Peters, Shannon B. Lim, Dennis R. Louie, Chieh-ling Yang, Janice J. Eng

**Affiliations:** 1 Department of Physical Therapy, Faculty of Medicine, University of British Columbia, 212 - 2177 Wesbrook Mall, Vancouver, BC V6T 1Z3 Canada; 2 Rehabilitation Research Program, Vancouver Coastal Health Research Institute, 4255 Laurel Street, Vancouver, BC V5Z 2G9 Canada; 3 Graduate Program in Rehabilitation Sciences, Faculty of Medicine, University of British Columbia, 212 – 2177 Wesbrook Mall, Vancouver, BC V6T 1Z3 Canada

**Keywords:** Functional near-infrared spectroscopy, Gait, Parietal cortex, Deoxyhemoglobin, Oxyhemoglobin

## Abstract

**Background:**

Experimental designs using surrogate gait-like movements, such as in functional magnetic resonance imaging (MRI), cannot fully capture the cortical activation associated with overground gait. Overground gait in a robotic exoskeleton may be an ideal tool to generate controlled sensorimotor stimulation of gait conditions like ‘active’ (i.e. user moves with the device) and ‘passive’ (i.e. user is moved by the device) gait. To truly understand these neural mechanisms, functional near-infrared spectroscopy (fNIRS) would yield greater ecological validity. Thus, the aim of this experiment was to use fNIRS to delineate brain activation differences between ‘*Active’* and ‘*Passive’* overground gait in a robotic exoskeleton.

**Methods:**

Fourteen healthy adults performed 10 walking trials in a robotic exoskeleton for *Passive* and *Active* conditions, with fNIRS over bilateral frontal and parietal lobes, and electromyography (EMG) over bilateral thigh muscles. Digitization of optode locations and individual T1 MRI scans were used to demarcate the brain regions fNIRS recorded from.

**Results:**

Increased oxyhemoglobin in the right frontal cortex was found for *Passive* compared with *Active* conditions. For deoxyhemoglobin, increased activation during *Passive* was found in the left frontal cortex and bilateral parietal cortices compared with *Active*; one channel in the left parietal cortex decreased during *Active* when compared with *Passive*. Normalized EMG mean amplitude was higher in the *Active* compared with *Passive* conditions for all four muscles (*p* ≤ 0.044), confirming participants produced the conditions asked of them.

**Conclusions:**

The parietal cortex is active during passive robotic exoskeleton gait, a novel finding as research to date has not recorded posterior to the primary somatosensory cortex. Increased activation of the parietal cortex may be related to the planning of limb coordination while maintaining postural control. Future neurorehabilitation research could use fNIRS to examine whether exoskeletal gait training can increase gait-related brain activation with individuals unable to walk independently.

## Background

In healthy adults, robotic exoskeletons are used for industrial purposes to reduce physical load with 10–80% of muscle activity reductions reported [1]. Though the influence of robotic exoskeletons on the musculoskeletal system is becoming known, the effect on the central nervous system is unknown. For individuals with stroke and spinal cord injuries, rehabilitation with robotic exoskeletons can positively affect gait outcomes, through standardized training and the option to increase therapeutic intensity [2–5]. Robotic exoskeleton use may impact neural activation; thus, it may be an ideal tool to generate controlled sensorimotor stimulation which may modulate neural activity through movement experience [6]. Indeed, brain-machine interfaces that detect gait initiation and intention are beginning to be integrated with robotic exoskeletons [7, 8]; however, these interfaces rely on electroencephalographic signals which are poorly localized because of volume conduction and variations in electrode placements.

For gait rehabilitation of individuals with neurological injury, conditions of gait within a robotic exoskeleton include ‘active’ (i.e. user moves with the device) and ‘passive’ (i.e. user is moved by the device) gait. Previous studies investigating surrogate gait-like movements using functional magnetic resonance imaging (fMRI, e.g. knee flexion/extension in supine) that compare passive to active stepping have inconsistent cortical activation findings. While one fMRI study found no-difference between passive and active conditions [9], another found increased activation in the sensorimotor, supplementary motor, and premotor cortex for active compared with passive stepping [10]. Further, two fMRI studies found increased activation for passive compared with active stepping in the: 1) prefrontal and posterior parietal area of the precuneus [10], and 2) cingulate and medial frontal areas, with motor response inhibition suggested as a possible mechanism to explain the increase in activation for passive stepping [11]. As fMRI tasks are completed in supine, these results have less ecological validity to overground gait as no postural control or maintenance of dynamic balance is required to prevent a fall [12]. These experimental designs cannot fully capture: a) the cortical activation associated with somatosensory feedback, or b) integration of that feedback together with motor planning, motor output, or motor response inhibition processes of overground gait. To truly understand these neural mechanisms, and how to better apply various conditions of robotic exoskeleton practice, neuroimaging during a dynamic, upright task would yield greater ecological validity.

Functional near-infrared spectroscopy (fNIRS) may offer a solution to this limitation. fNIRS is a lightweight, portable device ideal to record cortical brain activation during unrestricted movements, such as gait. It has improved signal localization over electroencephalography; as such, fNIRS can directly link localized neural activation during overground gait in a robotic exoskeleton. Using fNIRS, differences in cortical activation have been shown between standing and gait, fast and slow gait, as well as simple and complex gait [13]. However, to our knowledge, no experiments have yet used fNIRS to explore the cortical activation associated with various conditions of gait in a robotic exoskeleton.

When walking on a treadmill, increased activation of regions in the frontal lobe is shown with fNIRS (prefrontal cortex [PFC] [14], PFC/supplementary motor area [SMA] [15], SMA/primary motor cortex [M1] [16]). While the activation in the frontal lobe increases during gait [14–16], the brain activation related to gait is less clear posterior to M1 as recording of parietal regions beyond the primary somatosensory cortex is rare [16]. Yet, the posterior parietal cortex may be actively involved in feedback and feedforward control of upright gait [17–19]. Activation of the parietal cortex has been detected using fNIRS during dual-task gait, though these data were collected on a treadmill [20], which can generate different cortical activation than overground walking [21]. Parietal cortical activation may be even more likely in a robotic exoskeleton, however, this is unknown as experimental data to date has not recorded this far posteriorly.

While direct comparison of active and passive movement is difficult to standardize, many aspects of overground gait can be controlled using a robotic exoskeleton to allow comparison of cortical activation during active and passive movement. Thus, the primary aim of this experiment was to use fNIRS to delineate cortical activation differences between ‘*active’* and ‘*passive’* overground gait in a robotic exoskeleton. Our hypothesis was that for ‘*passive’* gait, the prefrontal cortex and posterior parietal regions would increase activation due to motor response inhibition, and that ‘*active’* gait would require greater activation of motor planning and sensorimotor cortical regions due to the need to control upright posture. Secondarily, we explored whether the posterior parietal cortex has a significant role in ‘*active’* and ‘*passive’* gait.

## Methods

Fourteen healthy adults (age = 34 ± 8 years; 7F, 7 M) volunteered to participate in this experiment approved by the Research Ethics Board at the University of British Columbia (H18–00400), with informed consent obtained from each participant in accordance with the Declaration of Helsinki. In addition, they had to be ≥19 years old, and healthy without gait impairment. Individuals were excluded if they could not safely fit into the exoskeleton (i.e., height < 5′0“ or >6’4”, weight > 220 lbs., leg length discrepancy > 0.5″ of the femur or > 0.75″ of the tibia, standing hip width > 18″, hip range of motion < 110°), or had neurological or medical conditions, pregnant, or any skin concerns at exoskeleton contact points.

### Experimental protocol

The protocol consisted of two sessions, separated by at least a one-day washout period, scheduled at the same time of day (±1 h). The purpose of the first session was to familiarize participants with the protocol, in particular, to practice the experimental conditions. At the first session only, researchers provided feedback on performance. No feedback on performance was given during the second session. The data from the second session was recorded, analyzed, and presented here.

The experimental task was to perform 10 walking trials over 10 m (+ 2 m ramp up/down, 14 m total) per each of: (1) *Passive*, (2) *Active* conditions. For the *Passive* condition, participants were instructed to be as relaxed as possible and to allow the exoskeleton to complete each step. For the *Active* condition, participants were instructed to work together with the exoskeleton to complete each step in accordance with the pre-programmed gait parameters. Details on the robotic exoskeleton settings are provided below. To allow for normalization of electromyographic (EMG) data, a control condition was collected where participants walked without the exoskeleton with EMG, matching the speed of the exoskeleton walking trials (further details below). The duration (seconds) of each walking trial for *Passive* and *Active* conditions was recorded to determine whether each walking condition was completed at a similar speed. A computer randomly determined whether participants started the testing with *Passive* or *Active* conditions so that half of the participants started with the *Passive* condition with the other half starting with the *Active* condition to counterbalance any potential carryover effects [22]. Prior to each walking trial, participants were asked to stand still with arms resting by their side, maintain a stable head position, and keep looking straight ahead.

#### Robotic exoskeleton

**Fig. 1:**
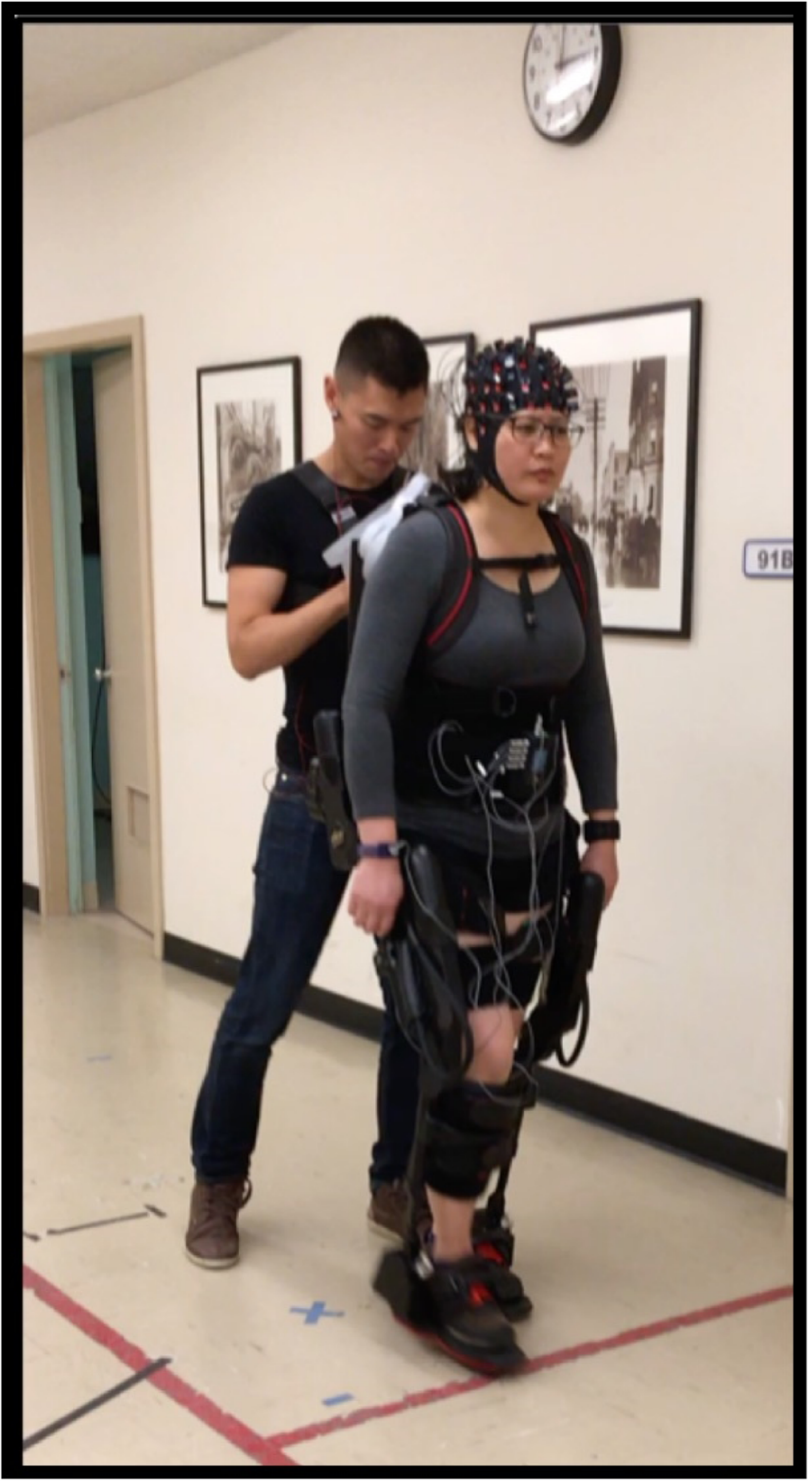
Representative participant and trained physical therapist. Functional Near-Infrared Spectroscopy (fNIRS) over bilateral frontal and parietal regions. Electromyography (EMG) of bilateral biceps femoris and rectus femoris

EksoGT (Ekso Bionics, Richmond, California, USA) is a commercially-available powered robotic exoskeleton that fits around the lower extremities and has electrically actuated motors at the hip and knee, and spring-loaded articulation at the ankle. The device can partially and fully power the legs through a cyclical walking motion. The battery and software for the system are housed in an attached backpack worn by the user and operated by a physical therapist (Fig. 1). The device is adjustable to fit many body sizes with modifiable gait settings so that assisted gait is matched to the user for parameters such as step length. For this experiment, the EksoGT stepping parameters were set to 14" or 14.5" for step length (longer step for taller people), 0.4″ for step height, and 1.2 s for swing time. The FirstStep walking mode was used for both Passive and Active, in which each step was triggered by the press of a button on a remote by the physical therapist. For Passive, the assistance provided by the exoskeleton was programmed to Maximum, in which the device will complete the same programmed stepping parameters regardless of user participation. For Active, the assistance was programmed to Adaptive, in which the device continually adapts assistance depending on the user’s effort to maintain the pre-determined limb trajectory. The exoskeleton and remote were operated by a registered physical therapist with 5 years of experience using the EksoGT, using headphones to listen to a metronome to ensure therapist-triggered stepping at 0.6 Hz for both exoskeleton conditions. For both Active and Passive conditions, the physical therapist operating the exoskeleton provided a verbal count down guided by the metronome during quiet standing over the 5 s prior to starting the walking condition.

A continuous dual-wavelength (760 and 850 nm) wearable fNIRS imaging system (NIRSport2, NIRx Medical Technologies LLC, Glen Head, NY, USA) assessed brain activation during each walking condition. To detect changes in blood concentration in the brain, the optical system consisted of 16 optode (light emitting diodes, LED) sources and 15 detectors separated by approximately 3 cm and affixed to the head using an EasyCap (EASYCAP GmbH, Germany) (Fig. 1). Eight short separation channels captured extraneous biological noise (e.g. cardiological and respiratory artifacts) to enable regression of these signals in the analysis. With this configuration, 54 channels including 8 short separation channels provided hemodynamic response estimations. The optodes were placed using the International 10–20 system centered over Cz in the mid-line of periauricular and nasion to inion measurements, similar to published recommendations [23, 24]. The optodes were situated over a large portion of the frontal and parietal lobes (Figs. 1, 5). All optodes were connected to a backpack which used local wi-fi to transmit the signal to a nearby laptop. The optodes were secured with multiple stabilizers to ensure minimal movement of wires during all conditions. Aurora fNIRS software (v. 5.02, NIRx Medical Technologies) recorded brain activation at 4.3597 Hz, and was used to manually place triggers associated with the start of each walking trial.

We took the additional step of localizing each optode’s location on every participant’s head with a 3D digitizer (Polhemus Patriot, Inc., VT). The digitized points were collected using the PHOEBE interface [25]. These x,y,z coordinates defined optode locations on the scalp relative to the nasion, inion, and periauricular points. These coordinates were used together with each individual’s brain anatomy (MRI T1), to estimate which brain regions the fNIRS channels were recording from.

#### MRI TI imaging

To co-register functional neuroimaging with structural data [26], MR acquisition of each participant’s brain was conducted at the University of British Columbia MRI Research Centre on a Philips Achieva 3.0 T whole body MRI scanner (Phillips Healthcare, Andover, MD) using an eight-channel sensitivity encoding head coil (SENSE factor = 2.4). A high-resolution T1-weighted anatomical scan (TR = 7.47 ms, TE = 3.65 ms, flip angle *Ɵ* = 6̊, FOV = 256 × 256 mm, 160 slices, 1 mm^3^ isotropic voxel) was acquired for each participant.

#### Electromyography (EMG)

EMG was collected to examine whether participants were able to produce the conditions asked of them (i.e. decreased muscle activity in the *Passive* condition compared with the *Active* condition). To determine level of muscle activity across conditions, after skin preparation, EMG pre-amplifier surface electrodes (SX230–1000) were placed bilaterally over the rectus femoris, and biceps femoris muscles to record muscle activity wirelessly at 1000 Hz using the Biometrics Ltd. DataLOG (Type No. MWX8, Software v 8.0, Newport, UK). All electrode placements followed the recommendations of SENIAM (https://www.seniam.org).

#### Walking kinematics

As variations in walking patterns can influence level of brain activation [27], and that the participant was given some active kinematic control during the Active condition, the *Xsens MVN Awinda™* (Xsens Technologies B.V., Culver City, California, USA) motion tracking system was used to examine whether knee kinematics were similar across conditions. It served as a control for whether brain activation could be attributed to potential kinematic differences between conditions. It is an inertial motion capture system which utilizes multiple sensors (gyroscope/accelerometer functionality), wireless communication, and sensor fusion algorithms to detect and measure joint angles. The inertial measurement units were placed over the sacrum, mid-thigh, mid-shank, and lateral feet with data collected using the Xsens MVN Analyze (v. 2019.0.0.0 build 1627, rev 78,643) program at 100 Hz.

### Data and statistical analysis

All statistical analyses were completed in IBM SPSS Statistics Viewer (v. 25).

fNIRS*:* The raw fNIRS signal was visually inspected before processing to identify movement artifacts, and checked after processing to ensure these artifacts were eliminated. fNIRS data were pre-processed using HomER2 [28]; all HomER2 functions and related parameters with are indicated by square brackets. After noisy channels were pruned [enPruneChannels function, SNRthresh = 2, dRange = 5e-04 to 1e+ 00, SDrange 0 to 45], raw intensity data were converted to optical density [hmrIntesnity2OD]. Changes in the absorption of near-infrared light was converted into relative concentration changes of oxy-hemoglobin (oxyHb) and deoxy-hemoglobin (deoxyHb) based on the modified Beer-Lambert law [29, 30]. Motion artifacts were identified by identifying a 0.5 s interval as motion artifact if the signal change was greater than 20 standard deviations for that channel [hmrMotionArtifactByChannel]. A wavelet transformation with 1.5 was chosen for the interquartile range, as recommended by Hocke et al. (2018) [hmrMotionCorrectWavelet] [31]. A low pass filter (0.15 Hz) was used [hmrBandpassFilt] prior to conversion of the optical density signal to concentrations using the partial pathlength factor of 6.0 for each wavelength [hrmOD2Conc] [32, 33]. Last, the hemodynamic response [hmrDeconvHRF_DriftSS] function was estimated using the time period − 5 to 30 s (− 5 to 0 as baseline during quiet standing coinciding with verbal count-down, 0 to 30 as gait task), with a regression of the short separation channels to remove extracerebral signals such as blood pressure waves, Mayer waves, respiration and cardiac cycles [34]. The chosen general linear model used an ordinary least squares [glmSolveMethod = 1], and consecutive sequence of gaussian functions [idxBasis = 1] as the type of basis function for the hemodynamic response estimation [time range for block average = − 5 30, paramsBasis = 0.5 0.5, rhoSD_ssThresh = 15, flagSSmethod = 1, driftOrder = 3] [35].

To retain the higher spatial resolution fNIRS affords, a channel-based statistical analysis was used, similar to Miyai et al. (2001) [36] and Koenraadt et al. (2014) [15], as opposed to a region of interest approach. Analysis was completed in two steps. The first step examined differences between quiet standing baseline (− 5 to 0 s) and walking task (0–30s) for each channel using paired *t*-tests, similar to other work [16]. The second step evaluated differences between *Passive* and *Active* conditions during walking with paired *t*-tests, only using the channels that were significantly different in the first step. To reduce the number of statistical tests at step two, these were the only channels that were entered into the task-based analysis. To control for multiple comparisons for the second step, the Benjamini-Hochberg adjustment was used (False Discovery Rate *q* = 0.01 [37–39], *p* < 0.05) which rank-orders *p*-values then adjusts for the number of comparisons performed [38, 40, 41].

MRI TI imaging*:* T1s were processed using FREESURFER’s (http://surfer.nmr.mgh.harvard.edu/) *recon-all* function to segment the T1 scans into head, white, and grey matter [42–44]. The output was entered into AtlasViewer [45] which registers head and probe with brain imaging information and overlaid with the digitized optode data obtained from PHOEBE. In AtlasViewer, the fNIRS channels, which were estimated as the midpoint between each digitized source-detector pair, were projected to individual MRIs. For each participant, a list of brain regions for each optode location was extracted using the Automated Anatomical Labelling atlas (AAL: Table 1).

**Table 1:** Significant channels and corresponding cortical regions based on individual anatomical MRI

Channel	Wavelength with Significant Task vs. Baseline	International 10–20 Optode Locations	Hemisphere	Cortical Region Based on Individual MRI (Total *n* = 14)^a^
2	oxyHb	F5-F3	L	Frontal (Middle *n* = 5, Inferior *n* = 9)
4	oxyHb & deoxyHb	F1-F3	L	Frontal (Superior *n* = 6, Middle *n* = 5, Superior/Medial *n* = 2); Mid cingulum (*n* = 1)
7	oxyHb	F2-Fz	R	Frontal (Superior *n* = 8, Superior/Medial *n* = 5); Mid Cingulum (*n* = 1)
17	oxyHb	FCz-FC1	L	Supplementary motor (*n* = 10); Frontal (Superior n = 3, Superior/Medial *n* = 1)
21	oxyHb	FC4-F4	R	Frontal (Inferior/Operculum *n* = 8, Middle *n* = 5, Inferior/Triangular n = 1)
26	oxyHb	C1-C3	L	Precentral (*n* = 12), Frontal (Superior *n* = 1, Middle *n* = 1)
28	oxyHb & deoxyHb	C1-CP1	L	Precentral (*n* = 6); Postcentral (*n* = 5); Paracentral (*n* = 3)
30	oxyHb	C2-FC2	R	Frontal (Superior *n* = 13, Middle *n* = 1)
33	oxyHb & deoxyHb	C2-CP2	R	Postcentral (*n* = 8); Precentral (*n* = 5); Supplementary motor (*n* = 1)
36	oxyHb	CP3-C3	L	Postcentral (*n* = 13); Parietal inferior (*n* = 1)
38	deoxyHb	CP3-P3	L	Parietal (Inferior *n* = 9, Superior *n* = 2); Angular (*n* = 3)
40	oxyHb &deoxyHb	CPz-Cz	R/L	Left Paracentral (*n* = 8); Right Paracentral (*n* = 3); Right Supplementary motor (*n* = 2); Right Postcentral (*n* = 1)
41	oxyHb &deoxyHb	CPz-CP1	L	Precuneus (*n* = 6); Postcentral (*n* = 4); Paracentral (*n* = 2); Parietal Superior (*n* = 2)
42	oxyHb	CPz-CP2	R	Precuneus (*n* = 6); Parietal Superior (*n* = 5); Paracentral (*n* = 2); Postcentral (*n* = 1);
43	deoxyHb	CPz-Pz	R/L	Left Precuneus (*n* = 8); Right Precuneus (*n* = 5); Right Parietal superior (*n* = 1)
52	deoxyHb	P2-CP2	R	Parietal (Superior *n* = 7, Inferior *n* = 3); Precuneus (*n* = 2); Postcentral (*n* = 2)
54	deoxyHb	P2-P4	R	Parietal (Inferior *n* = 6, Superior *n* = 4); Angular (*n* = 3); Occipital Sup (*n* = 1)

^a^Cortical region defined by the Automated Anatomical Labelling (AAL) atlas in AtlasViewer

Walking Duration*:* The durations of the 10 trials for the 2 conditions were averaged for each participant. To determine whether each walking condition was completed at a similar speed, the average time (s) was entered in a paired *t*-test.

EMG*:* Using custom Matlab (v. 2018b, MathWorks, Natick, MA) scripts, the raw EMG signals were detrended, bandpass filtered (20-400 Hz) with a 5th order Butterworth filter, rectified, and low-pass filtered (10 Hz). The mean amplitude of 10 bursts per trial for the *Passive* and *Active* were normalized to the mean amplitude of 10 bursts per trial for the control condition’s EMG, using the formula (*Passive*/control)*100 and (*Active*/control)*100. After normalization, paired t-tests for each muscle were completed.

Walking Kinematics: After files were batch re-processed in Xsens MVN Analyze (v. 2019.0.0.0 build 1627, rev 78,643), custom Matlab scripts extracted joint angle data. Specifically, maximal knee flexion was subtracted from the maximal knee extension angle for each trial, and averaged for each condition. Mean values were entered into 2 paired t-tests (1 for the right knee, 1 for the left knee).

## Results

Values are presented as mean ± standard deviation unless otherwise stated.

**Fig. 2:**
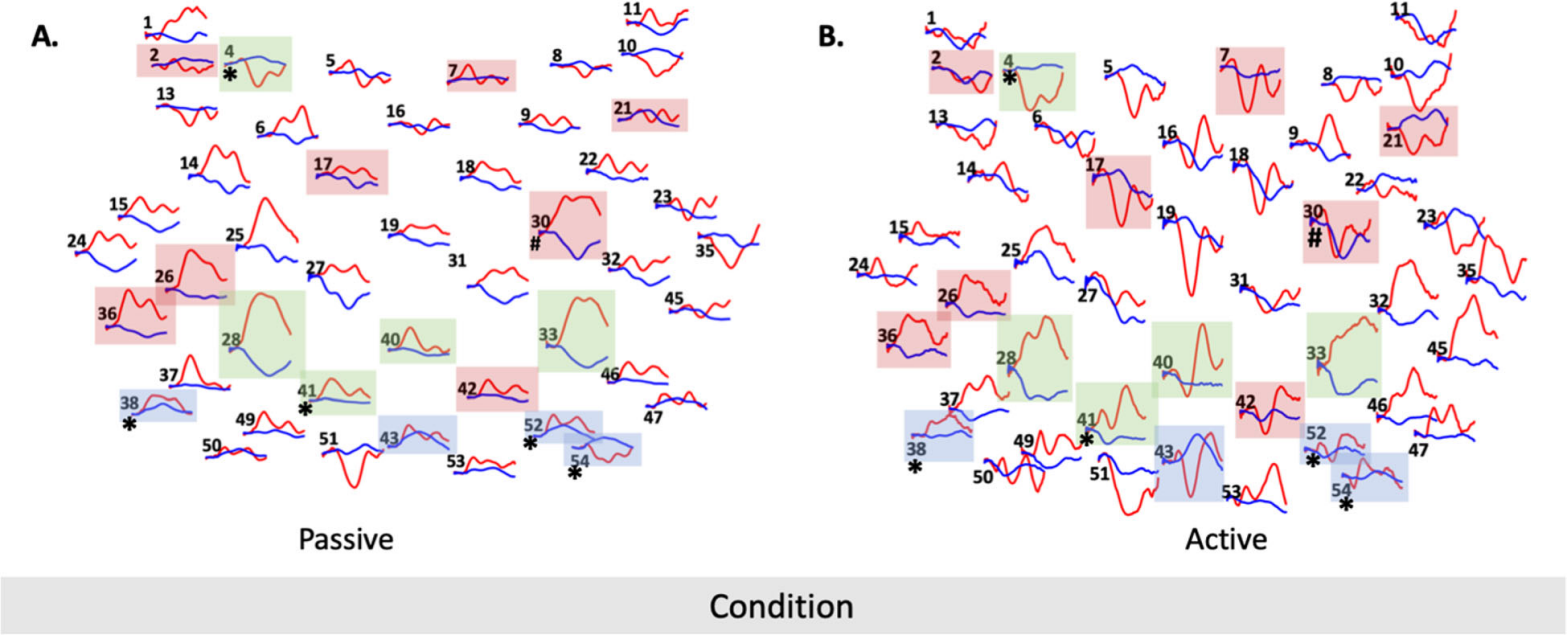
Hemodynamic response function plots for all channels with red traces indicating oxyHb and blue traces indicating dexoyHb. Red boxes indicate significant channels for baseline vs task for oxyHb, after the first step of analysis. Blue boxes indicate significant channels for baseline vs task for dexoyHb, after the first step of analysis. Green boxes indicate significant channels for baseline vs task for oxyHb and dexoyHb, after the first step of analysis. Hash symbol indicates oxy-hemoglobin and the asterisk indicates deoxy-hemoglobin significant differences between the Passive and Active conditions, after the second step of analysis

fNIRS: For each wavelength, Table 1 indicates the brain regions associated with the channels with significant activation during task compared with baseline. For the first step of analysis, 13 channels were significantly different during the walking task compared with quiet standing baseline for oxyHb, and 9 channels for deoxyHb (Fig. 2).

**Fig. 3:**
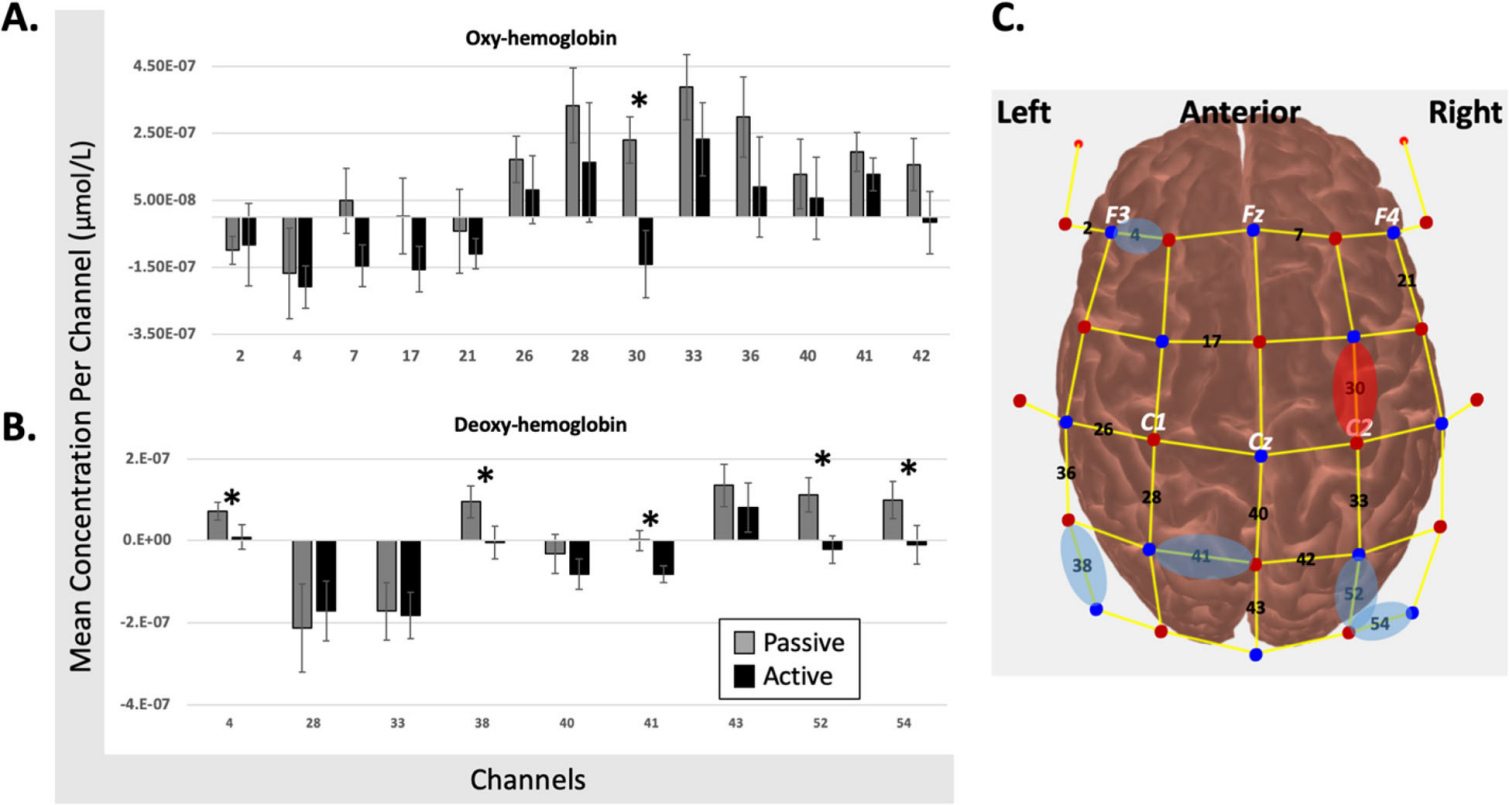
fNIRS results. Panels **a** (oxyHb results) and **b** (deoxyHb results) indicate the mean concentration (y axis) for each significant channel (x axis) after the first step of analysis. Error bars indicate standard error. Stars indicate significant differences between passive and active conditions after the second step of analysis. Panel **c** indicates the source/detector optode configuration with significant channels after the first step of analysis in black. Red oval indicates significant difference between passive and active conditions after the second step of analysis for oxyHb. Blue oval indicates significant difference between passive and active conditions after the second step of analysis for deoxyHb

After Benjamini-Hochberg correction, channel 30 increased oxyHb in *Passive* compared with *Active*; this activation was located in the right frontal cortex (superior gyrus for *n* = 13 participants, and middle gyrus for n = 1 participant; Fig. 3a, c). For deoxyHb, channels 4 (left frontal *n* = 13; left mid cingulum n = 1), 38 (left parietal *n* = 11, left angular *n* = 3), 52 (right parietal *n* = 10, right precuneus *n* = 2, right postcentral n = 2), and 54 (right parietal *n* = 10, right angular n = 3, right occipital n = 1) increased during *Passive*, and channel 41 (left precuneus *n* = 6, left postcentral *n* = 4, left paracentral n = 2, left parietal sup n = 2) decreased during *Active* (Fig. 3b,c).

It is worth noting that the error bars on Fig. 3a/b may indicate potential for Type 2 error, even after using a less conservative Benjamini-Hochberg correction of *q* = 0.1. While this is a possibility, the level of variability is similar to that reported in Koenraadt et al. (2014) (*n* = 11, 10 repetitions/condition) [15]. Further, methodologically the signal-to-noise for this study is likely increased with greater number of trials per condition and larger sample size than some gait studies using fNIRS [16, 27, 36]. So, it is conceivable that the variability reflects actual individual variation in brain activation associated with robotic exoskeleton assisted gait.

Walking duration: It took between 51.4 ± 5.2 (*Active*) to 52.8 ± 3.6 (*Passive*) seconds to walk 10 m, with no difference between conditions (*t* = 1.850, *p* = 0.087).

**Fig. 4:**
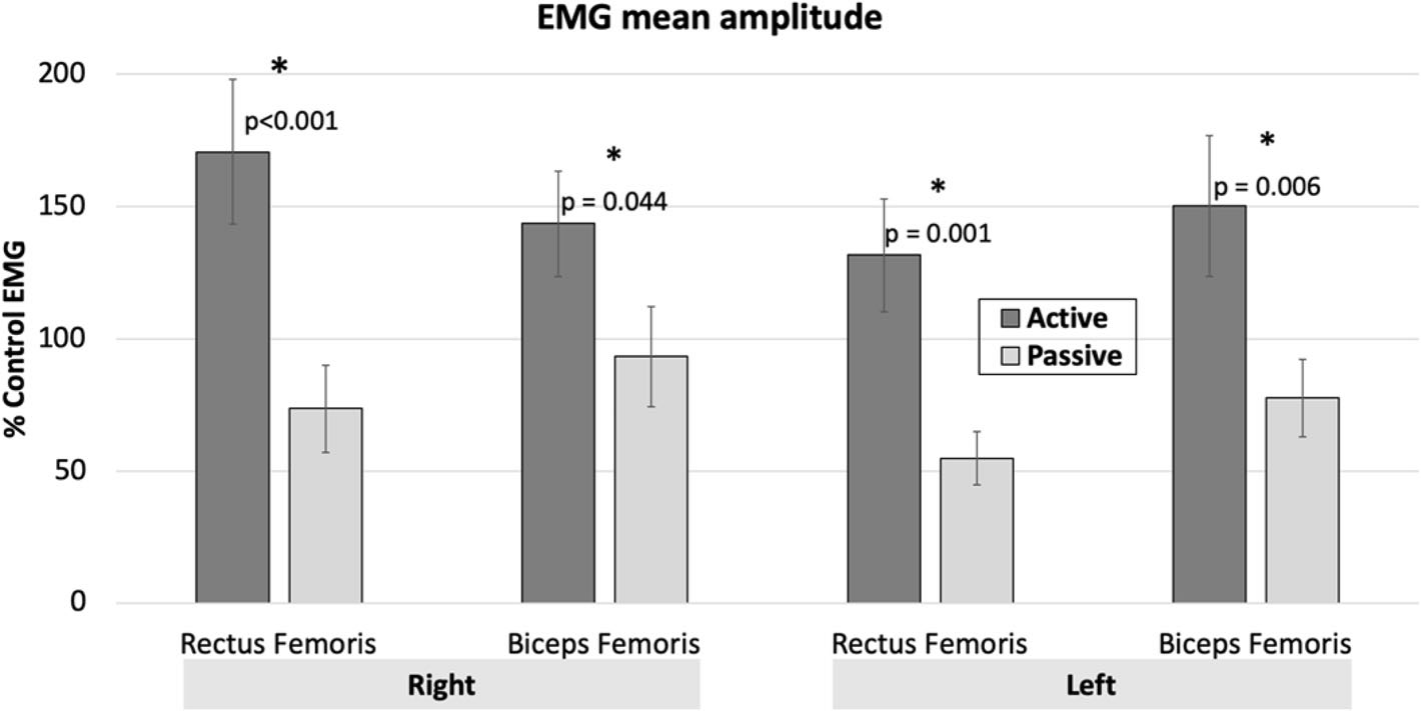
Mean EMG amplitude. Error bars indicate standard error. Star indicates significant differences between conditions

EMG: The normalized EMG mean amplitude was significantly higher in the *Active* condition compared with *Passive* for all four muscles (*t*_13_ ≤ 5.549, *p* ≤ 0.044; Fig. 4). This confirms that participants were able to produce the conditions asked of them.

### Walking kinematics

**Fig. 5:**
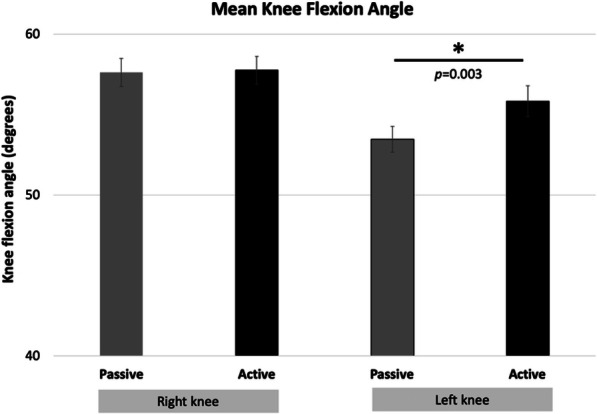
Walking Kinematic Results. Star indicates significant differences

Knee angles ranged from 53.5 ± 3.0 to 57.7 ± 3.2 degrees (Fig. 5). *Passive* was significantly larger than the *Active* condition by 2.4 degrees on the left (*t* = − 3.639, *p* = 0.003), with no difference between conditions on the right (*t =* − 0.029, *p* = 0.838).

## Discussion

We are the first to examine cortical activation of passive and active overground gait in a robotic exoskeleton. For our primary aim, in the passive condition, brain activation was present in regions associated with inhibition (channel 4), sensorimotor integration (channels 38/52/54), and sensorimotor output (channel 30; Table 1, Figs. 2, 3). In the active condition, decreased brain activation was present in regions associated with sensorimotor output (channels 30/41), and sensorimotor integration (channels 52/54) (Table 1; Figs. 2, 3). Compared with the active condition, passive gait showed increased activation of multiple bilateral parietal cortical regions together with the left frontal cortex. Active gait showed significant reduction of activation in sensorimotor and posterior parietal areas relative to the passive condition (channel 30/41, some reduction in channel 52/54; Table 1, Figs. 2, 3), contrary to our hypothesis which included regions with sensorimotor and motor planning functions (discussed below). For our secondary aim, there appears to be a large role for the posterior parietal cortex (PPC) during robotic exoskeleton walking, particularly in the passive condition which is further explored below.

### Changes in oxy- and deoxy-hemoglobin during robotic exoskeleton gait

A larger magnitude hemodynamic response in the sensorimotor, premotor, and prefrontal cortices is thought to depend on the complexity of the motor task, and whether the task is being learned [46, 47]. Though our experimental paradigm was novel for each participant, all practiced the tasks on a separate familiarization day. Thus, the observed increases in oxyHb and deoxyHb may reflect actual hemodynamic responses of passive and active exoskeletal gait. The majority of fNIRS studies during lower extremity tasks examine oxyHb alone, though some also examine deoxyHb [13, 40]. This may be as some research suggests that in fNIRS, interpretation of the deoxyHb signal is not straightforward [48, 49]. In an animal model, for a *decrease* in deoxyHb to be observed, both changes in cerebral blood flow and regional cerebral oxygen metabolic rate occur in venous blood [50]. To see an *increase* in deoxyHb, a change in venous dilation must occur without change to oxygen supply [50]. Based on this previous research, we chose to examine deoxyHb in our experiment as the deoxyHb signal may provide distinct information about brain activation unique from that of oxyHb [48]. Indeed, our experiment shows channels with significant changes in deoxyHb which inform our finding of parietal cortical involvement with robotic exoskeletal gait. While we may not be able to disentangle whether the increased deoxyHb was due to changes in oxygenation or blood volume, our main finding stands: the parietal cortex is important for robotic exoskeletal walking.

While oxyHb at channel 30 is reduced for the active condition, it is increased during the passive condition. Channel 30 is over the right frontal superior gyrus for *n* = 13 and over the frontal middle gyrus for *n* = 1 (Table 1), or the supplementary motor (SMA) and premotor (PMC) cortices, respectively. Previous research shows both increases and decreases in oxyHb in the frontal cortex during various walking conditions. In Koenraadt et al. 2014, decreased oxyHb is found with normal walking and precision stepping in the SMA [15]. Berger et al. 2019 showed decreased oxyHB for treadmill walking over SMA/PMC compared with the sensorimotor cortex [27]. In fMRI research, two studies found increased activation for passive compared with active stepping in prefrontal and posterior parietal area of the precuneus [10], and cingulate and medial frontal areas; motor response inhibition was proposed as a potential mechanism to explain the increased activity for passive stepping [11]. Taken together, the direction of activation within the frontal cortex is not consistently increased for active conditions, which may explain why our findings did not match our hypothesis. Relating this research to our results, the passive condition may require more cognitive planning resources than the active condition to inhibit the leg muscles. Conversely, the active condition is more similar to the daily walking healthy adults experience so less activation of regions associated with planning may be required.

### Upright postural control, locomotion, and the posterior parietal cortex

The PPC is involved with postural control [51], and may process somatosensation related to maintaining upright balance [52]. Manipulating a balance task to intensify demands on upright postural control increases activation of a network of regions within the frontal, central, and parietal cortices [53, 54]. Further, with an unexpected external balance perturbation, individuals with PPC lesions (stroke) demonstrate greater difficulty recruiting postural muscles of the lower limb in response, suggesting that the PPC is involved in the neural circuitry of reactive postural control, or that of maintaining the body’s center of gravity within the base of support following an unpredictable disturbance [18, 55]. Though our experimental design did not explicitly perturb participants, increased activation of the PPC during passive gait within a robotic exoskeleton may be related to a perceived need for a heightened overall state of reactive postural readiness. Likewise, if healthy adults use a robotic exoskeleton, such as in an industrial setting, to reduce physical burden to increase work output, a potential consequence may be greater processing load on some posterior parietal regions.

In humans, the PPC has multiple motor roles including sensorimotor integration and planning [56]. Structurally, neurons within the PPC project to the motor cortex and indicate the PPC has a role in modulating motor output [57]. Functionally, the PPC is a key region for sensorimotor integration [58]. In particular, Brodmann areas 5b and 7 within the PPC estimate an object’s location (particularly an obstacle) in the environment relative to the location of the body; this information is used to modify gait [19], and to coordinate limbs during locomotion [59]. Considering that our experimental paradigm did not involve obstacle navigation, perhaps the observed changes in activation relate to the planning function of the PPC [60]. The PPC is involved with planning limb-specific and limb-independent gait modifications [17], even when vision is temporarily occluded [61], and after unilateral lesion of Brodmann area 5 [62]. Within our experimental paradigm, increased activation of the parietal cortex during the passive condition (channel 38/52/54), and the decreased activation during the active condition (channel 41), may be associated with the planning of limb coordination while maintaining postural control.

### Future neurorehabilitation clinical trials

As many neurological conditions are associated with gait disorders, examining brain activation associated with gait and/or gait interventions are recommended with fNIRS [63, 64]. Passive movements are used in neurorehabilitation to maintain or improve mobility and range of motion, as well as to have individuals experience sensorimotor aspects of mobility that may be outside of their current abilities. Our finding of increased brain activation during passive gait, may indicate that passive gait practice within a robotic exoskeleton has potential for neuroplasticity, and by extension, motor recovery, though this requires testing in future research. Further, as oxyHb levels in the prefrontal and premotor cortices are sensitive to postural control, translation to clinical practice is being encouraged (see Gramigna et al. 2017 for review) [63]. However, the review did not identify research that examines regions more posterior to S1, like the posterior aspect of the parietal cortex. Gait rehabilitation is resource-intensive, requiring hours of physical therapists’ time and physical effort. Thus, integration of technologies like robotic exoskeletons could improve outcomes and resource utilization. The potential for using fNIRS to examine the cortical correlates of pathological gait is exciting. The important role of the parietal cortex in exoskeleton gait in healthy individuals is clear; however, future gait studies in patient populations should include experimental designs with fNIRS to capture the more posterior aspects of the parietal cortex to learn if this is true for pathological gait.

Additionally, fNIRS could be used to assess the effectiveness of novel interventions against current clinical practice. Furthermore, by exploiting its spatial resolution strength, fNIRS can be used to integrate known lesion properties in combined structural/functional studies. Another exciting potential is to examine whether exoskeletal gait training can increase gait-related brain activation with individuals who are unable to walk independently, if walking is an eventual clinical goal. This could exploit the increase in brain activation in the posterior parietal cortex and prefrontal regions, without requiring the patient to be able to walk independently overground, if the ability is not yet there. Furthermore, modulation of cortical activation could occur with neural recovery and a shift in gait training from passive to more active conditions. Thus, future work could explore how brain activation may change as a result of retraining gait using a robotic exoskeleton.

### Limitations

There are a few limitations to this work. First, fNIRS is confined to cortical examination. There are other subcortical brain regions that may contribute to robotic exoskeletal gait that cannot be examined with fNIRS (i.e central pattern generators, basal ganglia), meaning that fMRI continues to be a needed neuroimaging modality to learn about interactions between cortical and subcortical structures associated with gait. Second, EMG activation of the muscles of the shank was not completed, which may have affected analysis and interpretation of the fNIRS results. We made the decision not to record EMG over these muscles, as the ankle joint is not actuated within the EksoGT and so the majority of motor control is required around the knee. As indicated by the error bars in Fig. 4, for some individuals, the biceps femoris muscle during the passive condition is near or greater than 100% of the activation seen during the control EMG condition, which may be related to the function as an extensor muscle. However, this was not the case for all participants. The biceps femoris muscle may not be required to generate as much activation from a reduced requirement to eccentrically control the knee because the robotic exoskeleton provides external extensor support. Also, the EMG of other muscles within the quadriceps and hamstrings were not recorded, so it is unknown whether the muscle activation patterns of these muscles may have contributed to postural control. Third, the walking kinematics results indicate 2 degrees of larger knee motion on the left, however, this statistical difference may not be behaviourally meaningful.

## Conclusion

Varying the conditions of robotic exoskeleton gait modulates cortical activation, particularly for the parietal cortex. Delineating the cortical involvement during use of robotic exoskeletons is important, as the utility in industrial and rehabilitation contexts is increasing. Future work exploring the role of the parietal cortex in robotic exoskeleton gait could improve gait rehabilitation and foster expansion of its utility within industrial contexts.

## Data Availability

Anonymized data and materials supporting the conclusions of this article will be made publicly available at the University of British Columbia Digital Repository (https://circle.ubc.ca) beginning 18 months and ending 60 months following article publication.

## Abbreviations

deoxyHb: Deoxyhemoglobin
EMG: Electromyogram
F: Female
fMRI: Functional magnetic resonance imaging
fNIRS: Functional near-infrared spectroscopy
M: Male
M1: Primary motor cortex
MRI: Magnetic resonance imaging
oxyHb: Oxyhemoglobin
PFC: Prefrontal cortex
PPC: Posterior parietal cortex
SMA: Supplementary motor area
